# Prognostic significance of FOXM1 expression and antitumor effect of FOXM1 inhibition in synovial sarcomas

**DOI:** 10.1186/s12885-016-2542-4

**Published:** 2016-07-20

**Authors:** Akira Maekawa, Kenichi Kohashi, Masaaki Kuda, Kunio Iura, Takeaki Ishii, Makoto Endo, Tetsuya Nakatsura, Yukihide Iwamoto, Yoshinao Oda

**Affiliations:** Department of Anatomic Pathology, Graduate School of Medical Sciences, Kyushu University, 3-1-1 Maidashi, Higashi-ku Fukuoka, 812-8582 Japan; Departments of Orthopedic Surgery, Graduate School of Medical Sciences, Kyushu University, Fukuoka, Japan; Division of Cancer Immunotherapy, National Cancer Center Hospital East, Kashiwa, Japan

**Keywords:** Forkhead box M1 (FOXM1), Synovial sarcoma, Thiostrepton

## Abstract

**Background:**

Synovial sarcoma (SS) is a soft tissue sarcoma of unknown histogenesis. Most metastatic or unresectable cases are incurable. Novel antitumor agents and precise prognostication are needed for SS patients. The protein forkhead box M1 (FOXM1), which belongs to the FOX family of transcription factors, is considered to be an independent predictor of poor survival in many cancers and sarcomas, but the prognostic implications and oncogenic roles of FOXM1 in SS are poorly understood. Here we examined the correlation between FOXM1 expression and clinicopathologic and prognostic factors, and we investigated the efficacy of FOXM1 target therapy in SS cases.

**Methods:**

Immunohistochemical study of 106 tumor specimens was conducted to evaluate their immunohistochemical expression of FOXM1. An in vitro study examined the antitumor effect of the FOXM1 inhibitor thiostrepton and small interference RNA (siRNA) on two SS cell lines. We also assessed the efficacy of the combined use of doxorubicin (DOX) and thiostrepton.

**Results:**

Univariate and multivariate analyses revealed that FOXM1 expression was associated with poor prognosis in SS. The cDNA microarray analysis using clinical samples revealed that the expression of cell cycle-associated genes was correlated with FOXM1 expression. FOXM1 inhibition by thiostrepton showed significant antitumor activity on the SS cell lines in vitro. FOXM1 interruption by siRNA increased the chemosensitivity for DOX in both SS cell lines.

**Conclusion:**

FOXM1 expression is a novel biomarker, and its inhibition is a potential treatment option for SS.

**Electronic supplementary material:**

The online version of this article (doi:10.1186/s12885-016-2542-4) contains supplementary material, which is available to authorized users.

## Background

Synovial sarcoma (SS) is a soft tissue sarcoma of unknown histogenesis, occurring most frequently in adolescents and young adults. It is mainly classified into three histological subtypes: the biphasic type composed of both epithelial and spindle-cell components, the monophasic fibrous type composed of either an epithelial or spindle-cell component, and the poorly differentiated type [1]. SS has a genetic event, the t(X:18) translocation-mediated fusion of the SS18 gene on chromosome18q 11 to either SSX1, SSX2, or rarely SSX4 gene located on chromosome (p11.2;q11.2) [2]. The reported 5-year survival rates of patients with SS range from 64 to 77 % [3–6]. Most metastatic or relapsed diseases remain incurable, Efficacy of adjuvant chemotherapy in resected primary SS cases is still unclear [6]. Novel antitumor agents and precise prognostication are essential to improve the survival of SS patients.

The protein forkhead box M1 (FOXM1), a member of the FOX family of transcription factors, is widely expressed in embryonic tissues [7, 8]. Terminally differentiated nonproliferating tissues display relatively low levels of FOXM1 expression [9]. FOXM1 regulates a wide spectrum of tumor progression processes [10]. Increased levels of FOXM1 expression have been detected in many different types of human cancer [11–21] and sarcoma such as rhabdomyosarcoma [22], Ewing sarcoma [23], malignant peripheral nerve sheath tumor [24], and osteosarcoma [25, 26]. Silencing FOXM1 expression suppressed the proliferation of both cancer [16, 18, 22] and sarcoma cell lines [22, 26]. In various carcinoma cell lines, FOXM1 was also involved in resistance to chemotherapy drugs such as doxorubicin (DOX) [27], which is a frequently used antitumor agent against soft tissue sarcoma. The inhibition of FOXM1 may thus have the potential to be a therapeutic target for many malignancies. Both the prognostic impact of FOXM1 expression and the effectiveness of FOXM1 inhibition in SS remain to be clarified.

Here, we conducted a clinicopathologic and prognostic analysis of the FOXM1 expression in a series of 106 clinical specimens of SS, and a cDNA microarray analysis in 11 frozen samples. Using small interference RNA (siRNA), we then tested the involvement of FOXM1 in tumor progression and the acquisition of drug resistance. We also tested the efficacy of the combined use of DOX and FOXM1 inhibition (by thiostrepton and siRNA) in SS cell lines in vitro*.*

## Methods

### Patients and clinical information

We examined 106 SS patients registered in the Department of Anatomic Pathology, Graduate School of Medical Sciences, Kyushu University, Japan, between 1990 and 2014. Each tumor had been classified histologically into the monophasic fibrous, biphasic, or poorly differentiated type according to the most recent World Health Organization classification [28] including the examination of SS18-SSX1 and SS18-SSX2 fusion transcripts. The extents of necrosis and mitosis were evaluated according to the French Federation of Cancer Centers (FNCLCC) grading system [28]. For the staging of the primary tumors, the latest American Joint Committee on Cancer (AJCC) staging system was used [29]. Surgical margins were available in 49 patients (39 cases, wide marginal resection; 9 cases, marginal resection; 2 cases, intralegional resection).

We also analyzed the FOXM1 expression and EFS rate in 19 patients who had undergone pre- or/and post-operative chemotherapy. Eighteen of these patients had a wide margin; one patient underwent surgical resection, and one patient was treated with heavy ion irradiation. Most of the chemotherapy regimens were a single use of DOX or a combination of DOX and ifosfamide. This study was conducted in accordance with the principles embodied in the Declaration of Helsinki, and was approved by the Ethics Committee of Kyushu University (No. 26–49).

### Cell lines

We analyzed SYO-1 [30] was established by Dr. Kawai and HS-SY-II [31] was established by Dr. Sonobe as synovial sarcoma cell lines. These cell lines were authenticated by confirming the expression of pathognomonic SS18-SSX fusion genes by reverse transcriptase polymerase chain reaction (RT-PCR) in October 2012. All cell lines were cultured in Dulbecco’s modified Eagle’s medium (DMEM) supplemented with 10 % fetal bovine serum (FBS) plus penicillin.

### Drugs

Doxorubicin (DOX) was obtained from Cell Signaling Technology (Tokyo), and Thiostrepton was obtained from Millipore/EMD (Billerica, MA, USA). Both drugs were dissolved in DMSO (Sigma-Aldrich, St. Louis, MO) and were used at the indicated concentrations.

### Detection of fusion gene transcripts

We performed an SS18-SSX fusion assay based on the reported primers [32] that specifically amplify the fusion gene transcripts of SS18-SSX1 and SS18-SSX2. Each PCR product (5 μL) was loaded onto a 2 % agarose gel with ethidium bromide and visualized under UV illumination. The PCR products were also evaluated by direct sequence analysis using the Big-Dye terminator method (version 1.1; Applied Biosystems, Foster City, CA) to confirm the breakpoints of fusion transcripts.

### Immunohistochemical study

All 106 formalin-fixed, paraffin-embedded specimens were cut at 3 μm. Antigen retrieval was carried out by boiling the slides with Target retrieval solution (TRS; Dako, Carpinteria, CA). The primary antibody was monoclonal anti-human FOXM1 antibody (R&D Systems, Minneapolis, MN) diluted at 1:300. All immune complexes were visualized by the EnVision™ System Detection system (Dako).

We used biopsy specimens for the evaluation of FOXM1 expression if the patients received pre-operative chemotherapy. For FOXM1, immunoreactivity was defined as cells showing nuclear staining with/without cytoplasmic staining patterns in the tumor tissue with minimal background staining. Tumors with a strong staining intensity in >10 % of the tumor cells were recorded as having positive immunoreactivity for FOXM1 based on a reported method [11, 12]. The serial sections were also immunostained with anti-Ki-67 antibody (M 7240, 1:100; Dako Glostrup, Denmark) using the standard procedure. The Ki-67-labeling index was calculated as described [33].

### Gene expression profiling of cDNA micro array

We conducted cDNA micro array analysis in 11 frozen samples obtained from primary SS cases. For the Oligo DNA microarray analysis, 3D-Gene Human Oligo chip 25 k (Toray Industries, Tokyo) was used (25,370 distinct genes). For efficient hybridization, this microarray has three dimensions and is constructed with a well as the space between the probes and cylinder-stems with 70-mer oligonucleotide probes on the top. Total RNA was labeled with Cy5 using the Amino Allyl MessageAMP™ II aRNA Amplification Kit (Applied Biosystems). The Cy5-labeled aRNA pools and hybridization buffer, and hybridized for 16 h.

The hybridization was performed using the supplier’s protocols (www.3d-gene.com). Hybridization signals were scanned using a ScanArray Express Scanner (PerkinElmer, San Jose, CA), and processed by GenePixPro software, ver. 5.0 (Molecular Devices, Sunnyvale, CA). The raw data of each spot was normalized by subtraction with a mean intensity of the background signal determined by all blank spots’ signal intensities of 95 % confidence intervals (CI). The signals detected for each gene were normalized by the global normalization method (the median of the detected signal intensity was adjusted to 25). Genes correlated with *FOXM1* were extracted by the hierarchal clustering method. We defined “correlate” as a correlation coefficient (CC) > 0.828. We also conducted a gene ontology (GO) analysis using the Gene Ontology Consortium (http://geneontology.org/).

### siRNA

Both SYO-1 and HS-SY-II cells were transfected with On-Target plus Smart Pool siRNAs *FOXM1* (Dhamacon, CO, USA) and On-Target plus Non-targeting Pool (Dhamacon, CO, USA) as a control, using Lipofectamine RNA imax (Invitrogen, MA, USA) according to the manufacturer’s protocols. The introduction of the siRNA for FOXM1 was confirmed by qRT-PCR and immunoblotting.

### TaqMan PCR to detect mRNA quantity of *FOXM1*

Total RNA was extracted using miRNeasy Mini kit (Qiagen). Five micrograms of RNA from each sample were reverse-transcribed using Quantitect Reverse Transcription Kit (Qiagen) in order to prepare first-strand cDNA. We performed a quantitative RT-PCR for *FOXM1* and analyzed using TaqMan assay reagents (FOXM1 Hs00170471_m1.; GAPDH Hs99999905_m1.; Applied Biosystems) and an ABI Prism 7700 Sequence Detection system (Applied Biosystems). RNA was obtained from 23 frozen samples and cell lines, using Qiagen mi RNA extraction kit (Qiagen, Venlo, Netherlands). The RNA extraction and PCR reaction were carried out according to the manufacturer’s protocol. The obtained data were standardized using the data of the housekeeping gene GAPDH. All of the reactions for each sample were performed in at least triplicate. The data were averaged from the values obtained in each reaction.

### Western blot

The cells were washed twice with ice-cold phosphate-buffered saline (PBS), scraped, and collected in a microcentrifuge tube. Whole cell lysates were prepared from the cell lines. Anti-FOXM1 (1:200 dilution) antibody (R&D Systems). Anti-human actin mouse monoclonal antibody (1:5000; Millipore) was used as a loading control. The subsequent Western blot procedure was performed as described [33].

### Cell viability

Cell viability was assessed by an MTT assay using the Cell Counting Kit 8 (CCK-8, Dojindo Molecular Technologies, Rockville, MD) according to the manufacturer’s instructions and as described [33]. The absorbance at 450 nm was measured by a microplate reader (Model 680, Bio-Rad Laboratories) by spectrophotometry at 450 nm.

### Drug treatment and cell proliferation assay of the transfected cell lines

After 24-h siRNA transfection, the transfected cells were seeded at 5000 cells per well in 96-well plates. For the chemosensitivity assay, various concentrations of DOX were added to the medium after 12-h incubation. After another incubation for 72 h, the number of viable cells in each well was measured.

For the proliferation assay, the number of viable cells in each well was measured at 36, 48, 72, and 96 h after transfection. Assays were conducted in triplicate and were repeated at least three times in separate experiments.

### Drug treatment and cell proliferation assay

SYO-1 and HS-SY-II cells were plated on 96-well plates at a concentration of 5000 cells per well in serum-containing growth medium. After a 12-h incubation, cells were treated with carrier alone (0.01 % DMSO) as non-treated control or with various concentrations of DOX, thiostrepton, or thiostrepton + DOX for another 72 h. The resulting data are reported as the percentage of cell viability in comparison to that of the respective non-treated control group (100 %). Assays were conducted in triplicate and were repeated at least three times in separate experiments.

### Statistical analysis

We used the chi-square test and the *t*-test as appropriate to evaluate associations between two variables. The Steel-Dwass multiple comparison test was applied to compare the data of more than two groups. The survival correlations are illustrated with Kaplan-Meier curves, and survival analyses were performed using the log-rank test. In the multivariate analysis, a Cox proportional hazards model was used to independent examine factors. Two-sided *P*-values <0.05 were considered significant.

## Results

### Prognostic significance of FOXM1 expression in synovial sarcoma patients

Survival data were available for overall survival (OS) in 103 patients, who were followed-up from 1 to 278 months (median, 85 months). The 5-year OS rate was 62 %. Data were available for event-free survival (EFS) in 70 patients, who had a follow-up ranging from 4 to 278 months (median, 81 months) and whose 5-year EFS rate was 56 %.

**Fig. 1:**
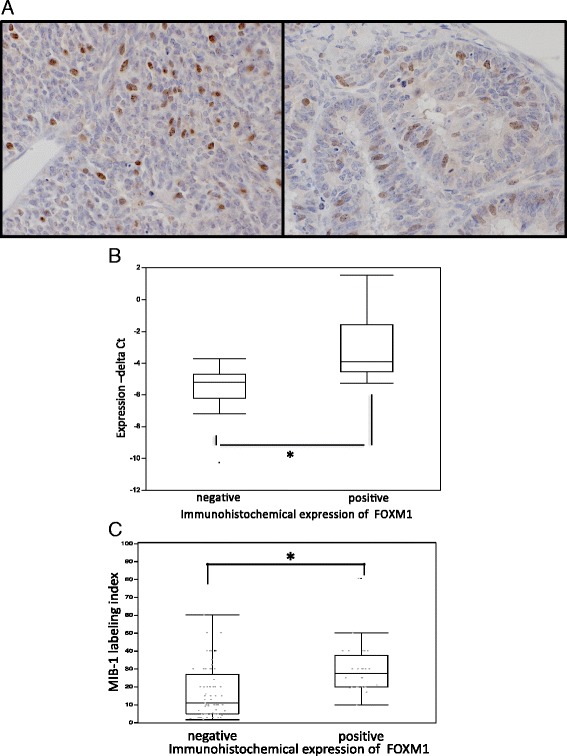
**a** Immunohistochemical results for FOXM1: Monophasic fibrous type (*left*) and biphasic type (*right*). Immunostaining for antibody was recognized in the nuclei. **b** Quantitative RT-PCR and immunohistochemical stain for FOXM1 in clinical samples. The RT-PCR values are plotted as: 1 × (cross threshold [Ct] FOXM1 − Ct GAPDH). High Ct values indicate high gene expression, and vice versa. The results are the means ± SD. *P < 0.05 by *t*-test. **c** Correlation of FOXM1 expression and MIB-1 labeling index in clinical specimens. The MIB-1 labeling index was significantly high in the FOXM1 expression cases. The results are means ± SD. *P < 0.05 by *t*-test

Immunohistochemically, the positive expression of FOXM1 was recognized in 28 of the 106 SS cases. SS cells showed nuclear staining for FOXM1 antibodies (Fig. 1a). The mRNA expressions of the samples that were immunohistochemically positive for FOXM1 showed a significantly higher mean cross threshold (mean, −2.95 ± 2.20) compared to the immunohistochemically negative expression samples (mean, −5.64 ± 1.63; *P* = 0.002) (Fig. 1b). In addition, the MIB-1 labeling index was significantly higher in the FOXM1 expression cases (positive 29.3 ± 13.5 vs. negative 16.9 ± 14.0, *P* = 0.0002) (Fig. 1c).

**Fig. 2:**
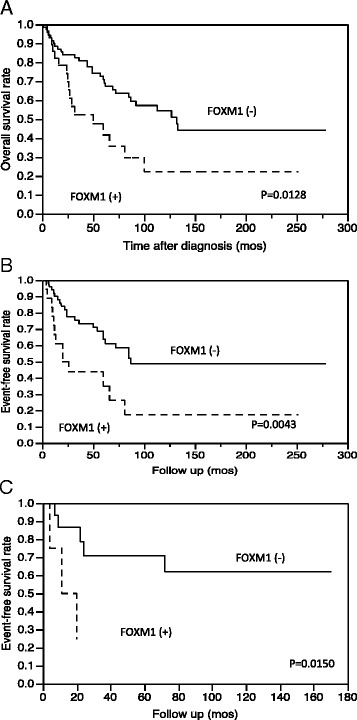
Kaplan-Meier survival curves for the patients’ overall survival (OS) and event-free survival (EFS) according to the results of the immunohistochemical study for FOXM1: (**a**) OS, (**b**) EFS, and (**c**) EFS for the 19 patients treated with chemotherapy

The clinicopathologic data and the results of the survival analysis of all 106 patients are summarized in Table 1. We evaluated the correlations between the immunohistochemical results and over-all (OS) or event-free (EFS) survival. Immunopositivity for FOXM1 was found to be a significant risk factor for adverse prognosis (OS and EFS). The Kaplan-Meier survival curves for OS and EFS are shown in Fig. 2a,b. Among the 19 patients treated with chemotherapy, the 3 FOXM1 expression cases had poor prognoses in EFS (Fig. 2c).

**Table 1:** Clinicopathologic parameters, FOXM1 expression and survival analysis

Variable	No. of patients	Analyzed groups	*P*-value		FOXM1		
			OS	EFS	Positive	Negative	*P*-value
Sex							
Male	44				13	31	
Female	62	Male vs. female	0.0028*	0.3047	17	45	0.811
Age							
< 20	19				4	15	
≥ 20	87	20 < vs. ≥20	0.0189*	0.2499	26	61	0.4279
Chemotherapy							
Yes	25				6	19	
No	25	Yes vs. No	0.3431	0.8037	4	21	0.4783
N.A.	56						
Fusion gene type							
SS18-SSX1	30				11	19	
SS18-SSX2	14	SSX1 vs. SSX2	0.6271	0.8581	3	11	0.3736
N.A.	62						
Depth							
Superficial	13				5	8	
Deep	91	Deep vs. Superficial	0.4441	0.9057	25	66	0.4243
N.A.	2						
Size,cm							
< 5	40				8	32	
5≥	62	<5 vs. 5≤	0.0012*	0.0335*	22	40	0.0885
N.A.	4						
Histological subtype							
Mono	69				22	47	
Bi	26	Mono vs. bi	0.4225	0.9701	3	23	0.0335*
Poor	3				1	2	
Undetermined	8						
Necrosis							
None	56				13	43	
≤ 50 %	26	Necrosis (+) vs. (−)	<0.001*	0.0012*	9	17	0.1526
> 50 %	15				6	9	
N.A.	9						
Mitotic count							
≥ 10/10HPF	70				12	58	
< 10/10HPF	31	≥10 vs. <10/10HPF	0.0344*	0.0056*	17	14	0.0002*
N.A.	5						
AJCC stage							
II	39	II vs. III	0.0304*	0.3734	11	28	0.72
III	44	III vs. IV	<0.001*	-	14	30	0.6579
IV	13				5	8	
N.A.	10						
FNCLCC							
2	69				18	51	
3	23	2 vs. 3	<0.001*	<0.001*	10	13	0.123
N.A.	14						
FOXM1							
Positive	30						
Negative	76	Positive vs. negative	0.0128*	0.0043*	-	-	-

*AJCC* American Joint Committee on Cancer, *Bi* biphasic synovial sarcoma, *EFS* event-free survival, *FNCLCC* French Federation of Cancer Centers, *HPF* high-power fields, *Mono* monophasic synovial sarcoma, *Poor* poorly differentiated synovial sarcoma, *NA* not available, *OS* overall survival

*P < 0.05 by log-rank test or chi-square test

The following clinicopathologic variables were also revealed to be significantly associated with poor prognosis: large tumor size (>5 cm), the presence of tumor necrosis, high mitotic activity (>10/10 HPF), advanced AJCC stage (II vs. III and III vs. VI), sex (female) and age (>20 year). The associations of the clinicopathological parameters with FOXM1 are shown in Table 1. FOXM1 was significantly associated with the histological subtype (monophasic fibrous variant) and high mitotic activity (>10/10 HPF).

We also conducted a multivariate analysis for FOXM1 with clinicopathologic variables adjusted by sex, age and AJCC surgical stage (II, III and IV) that were related to poor prognosis in the univariate analysis. We excluded tumor depth, tumor size, mitotic count, necrosis and FNCLCC histological grade, because the AJCC surgical stage is derived from these. The multivariate analysis revealed that FOXM1 expression and AJCC staging are significantly correlated with overall survival (Table 2).

**Table 2:** Multivariate analysis for immunohistochemical and clinicopathological parameters

Multivariate analysis	
Variable	*P*-value (overall survival)
AJCC staging	<0.0001*
age (20 < vs ≥20)	0.2819
Sex	0.5497
FOXM1	0.0302*

*P < 0.05 by Cox proportional hazards model

### Gene expression analysis in clinical samples

We conducted a cDNA microarray analysis in 11 frozen samples, and the clustering analysis showed that 32 gene expressions were significantly correlated with *FOXM1* expression (CC >0.828). They are listed in Table 3. The gene ontology (GO) analysis revealed that GO terms that were involved in the cell cycle or mitotic process were enriched in the cluster including *FOXM1* (Table 4). Raw data from the microarray analysis are available on the website of the Gene Expression Omnibus (accession no.GSE65532, http://www.ncbi.nlm.nih.gov/geo/).

**Table 3:** cDNA microarray data analysis: hierarchal cluster of gene expressions correlated with FOXM1 in 11 frozen SS samples (correlation coefficient > 0.828)

Gene symbol	Description
FBXO5	F-box only protein 5 (Early mitotic inhibitor 1)
TTK	Dual specificity protein kinase TTK)(Phosphotyrosine picked threonine-protein kinase)(PYT)
CENPM	Centromere protein M (CENP-M)(Proliferation-associated nuclear element protein 1)
KIF11	Kinesin-like protein KIF11 (Kinesin-related motor protein Eg5)
SGOL2	Shugoshin-like 2 (Tripin)
RBM12	Copine-1 (Copine I)
GINS2	DNA replication complex GINS protein PSF2 (GINS complex subunit 2)
CLSPN	Claspin (hClaspin)(Hu-Claspin)
ASF1B	Histone chaperone ASF1B (Anti-silencing function protein 1 homolog B)(hAsf1)(hAsf1b)
PRR11	Proline-rich protein 11
BIRC5	Baculoviral IAP repeat-containing protein 5 (Apoptosis inhibitor survivin)(Apoptosis inhibitor 4)
GTSE1	G2 and S phase-expressed protein 1 (B99 homolog)
C13orf3	Uncharacterized protein C13orf3
DIAPH3	Protein diaphanous homolog 3 (Diaphanous-related formin-3)(DRF3)
C16orf75	OB DNA-binding domain-containing protein C16orf175
NCAPD3	Condensin-2 complex subunit D3 (Non-SMC condensin II complex subunit D3)(hCAP-D3)
LMNB2	Lamin-B2
KIF23	Kinesin-like protein KIF23 (Mitotic kinesin-like protein 1)(Kinesin-like protein 5)
C15orf23	Putative TRAF4-associated factor 1
NCAPH	Condensin complex subunit 2 (Non-SMC condensin I complex subunit H)(Barren homolog protein 1)
CDCA4	Cell division cycle-associated protein 4 (Hematopoietic progenitor protein)
NUF2	Kinetochore protein Nuf2 (hsNuf2)(hNuf2)(hNuf2R)(Cell division cycle-associated protein 1)
HCAP-G	Condensin complex subunit 3
TOP2A	TOP2A_HUMAN Isoform 2 of P11388
CDCA3	Cell division cycle-associated protein 3 (Trigger of mitotic entry protein 1)(TOME-1)
PBK	Lymphokine-activated killer T-cell-originated protein kinase
NCAPG2	Condensin-2 complex subunit G2
CCNA2	Cyclin-A2 (Cyclin-A)
ZWINT	ZW10 interactor
CENPN	Centromere protein N (CENP-N)
KNTC2	Kinetochore protein NDC80 homolog
RFC5	Replication factor C subunit 5 (Activator 1 subunit 5)

**Table 4:** Gene ontology analysis: list of GO terms that were enriched in the cluster including FOXM1 compared with reference genes (*P* < 5 × 10 − 14)

Term	Sample frequency (27 genes)	Background frequency (21,804 genes)
Mitotic cell cycle (GO:0000278)	21	763
Cell cycle (GO:0007049)	21	1251
Cell cycle process (GO:0022402)	19	972
Mitotic cell cycle process (GO:1903047)	17	685
Nuclear division (GO:0000280)	15	420
Organelle fission (GO:0048285)	15	446
Cell cycle phase (GO:0022403)	13	287
Biological phase (GO:0044848)	13	291
Mitotic nuclear division (GO:0007067)	13	317
M phase (GO:0000279)	11	216
Mitotic M phase (GO:0000087)	11	216

### Antitumor effect of FOXM1 knockdown in SS cell lines

**Fig. 3:**
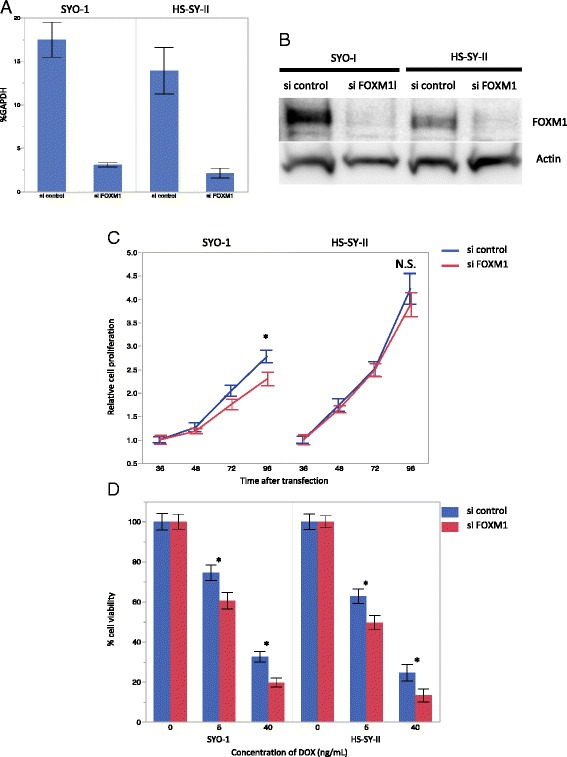
Proliferation and chemosensitivity assay results in SS cell lines with FOXM1 knockdown. **a** The cell lines were transduced with FOXM1 siRNA or a nontargeting control. The real-time quantitative PCR for FOXM1 showed a reduction in FOXM1 transcript at 24 h after transfection. **b** Western blotting demonstrated that the cell lines transduced with FOXM1 had significantly reduced levels of FOXM1 protein at 48 h after transfection. **c** Cell lines with FOXM1 siRNA compared to nontargeting control. Significantly decreased proliferation was recognized in the SYO-1 cells at 96 h after transfection. **d** SiRNA targeting FoxM1 transfected cells had higher sensitivity for DOX, compared with the control. Data are presented as mean ± SD for three independent experiments. *P < 0.05 by by *t*-test. N.S., not significant

We knocked down FOXM1 in both cell lines by using siRNA. The interruption of FOXM1 was confirmed by Western blotting and TaqMan PCR in both cell lines (Fig. 3a,b). Reduced cell proliferation was recognized only in the SYO-1 cells, not in HS-SY-II (Fig. 3c). Increased sensitivity for DOX was observed in both cell lines by FOXM1 interruption (Fig. 3d).

### Antitumor effect of thiostrepton and DOX for SS cell lines

**Fig. 4:**
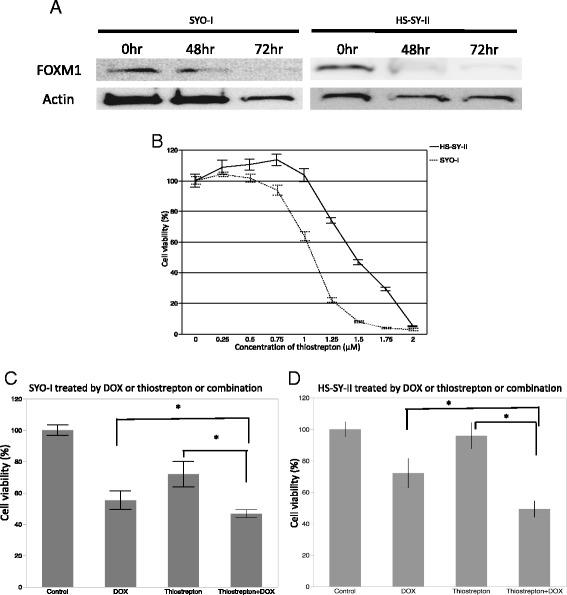
Thiostrepton reduced FOXM1 expression in the SS cell lines, producing diminished cell viability. **a** SS cell lines treated with 1 μM thiostrepton for 48 h and 72 h showed decreased FOXM1 protein on Western blots. **b** Treatment of SS cell lines with increasing quantities of Thiostrepton for 72 h resulted in reduced numbers of viable cells compared to diluent controls. **c** and **d** Proliferation of SS cell lines treated with 1 μM thiostrepton, 5 ng/mL DOX, or their combination. The cell lines treated with the combination of both drugs showed lower proliferation than those treated with each drug individually (**c**: SYO-I, **d**: HS-SY-II). *P < 0.05 by Steel-Dwass multiple comparison test

Compared to the untreated controls, decreased FOXM1 expressions were recognized in treated tumor cells by Western blotting (Fig. 4a). Thiostrepton dose-dependently inhibited the cell proliferation for both the SYO-1 and HS-SY-II SS cell lines (Fig. 4b). We also evaluated the effect on the proliferation of cell lines treated with thiostrepton, DOX, or their combination. We observed that the cell lines treated with the combination of both drugs showed lower proliferation than those treated with either drug individually (Steel-Dwass multiple comparison test, *P* < 0.05) (Fig. 4c and d). and TaqMan PCR in both SS cell lines treated with thiostrepton (Additional file 1: Figure S1).

## Discussion

The expression of FOXM1 in clinical specimens has been reported to be an adverse prognostic factor in many malignancies [11, 15–17, 19–21, 24]. In the present study’s univariate analysis, FOXM1 expression was revealed to be correlated with poor prognosis for OS and EFS among the SS patients treated with chemotherapy, and the multivariate analysis adjusted for surgical stage, sex and age showed that FOXM1 expression was an independent prognostic factor. Among the clinicopathological factors, high mitotic activity was strongly correlated with overexpression of FOXM1. Immunohistochemically, the MIB-1 labeling index was significantly high in the FOXM1 expression cases.

The cDNA microarray showed that 32 gene expressions were significantly correlated with *FOXM1* expression in clinical samples of SS. The GO analysis revealed that many of these genes are involved in the cell cycle and mitosis. Three genes (CCNA2 [34], KIF23 [35] and CDCA3 [36]) involved mainly in controlling late cell-cycle events in the G2 and M phases were among this group. These three genes have the CHR (cell cycle genes homology region) element in their promoter lesion [37], and FOXM1 controls cell cycle-dependent gene expression through CHR elements [38]. The CHR, typically located at or close to the transcriptional start site of a cluster of genes at the G2-M transition, is coordinated through promoter elements bounded by the dimerization partner, RB-like, E2F and multi-vulval class B (DREAM) and Myb-MuvB (MMB) transcriptional regulatory complexes [38].

Topoisomerase IIα and survivin (BIRC5), which are mainly involved in controlling early-phase cell-cycle events, was also shown to correlate with FOXM1 by cDNA microarray. Topoisomerase IIα plays a role in mitotic chromosome condensation and segregation, creating double-strand breaks in DNA [39]. In a mouse lung tumogenesis model, FOXM1 directly bound to the topoisomerase IIα promoter region [40]. Oda et al. reported that the survival of SS patients with a high expression of topoisomerase IIα was worse than that of SS patients with a lower expression [41].

Survivin forms a complex with chromosome passenger proteins Aurora B kinase and inner centromere protein (INCENP), where it plays a critical role in the localization of the Aurora B kinase-INCENP complex to the inner chromosomal region of centromeres at the early stages of mitosis [42]. Interruption of FOXM1 reduced the survivin expression in leukemia [21] and osteosarcoma [25] cell lines and inhibited cell-cycle progression. Survivin also associated with DNA damage response, it may facilitate recruitment of repair proteins at sites of DNA damage and inhibition of survivin mediate the increase chemosensivity for DOX in leukemia cell line [43].

DOX is routinely available for sarcoma treatment in many countries. The first-line chemotherapy for advanced, metastatic or nonresectable soft tissue sarcoma is typically based on DOX as a single agent or in combination with a second drug such as ifosfamide [44].

The results of the present cDNA microarray analysis supported our clinical and pathological finding that FOXM1 expression was correlated with high mitotic activity, a high MIB-1 labeling index and poor prognosis in SS patients. These findings indicated that FOXM1 is reliable biomarker for adverse prognosis in SS patients.

FOXM1 interruption by siRNA caused a reduction in cell proliferation, significantly so in the cell line SYO-1. FOXM1 interruption caused decreasing viability treated with DOX in both the SYO-1 and HS-SY-II cells. DOX treatment of cancer cells created double-stranded DNA breaks, and DNA repair genes were induced to rescue the cells from the DNA damage. FOXM1 regulates survivin and other DNA repair genes [45] (XRCC1 and BRCA2) and is involved in chemoresistance via a DNA repair pathway. Other investigators reported that the interruption of FOXM1 expression in breast cancer cells sensitized the cells to DOX [46].

DOX induces acute and chronic toxicities, and treatment options are needed to reduce the dosages of DOX and enhance its therapeutic efficacy [44]. Here we demonstrated that FOXM1 expression has important roles in cell proliferation and chemoresistance in SS cell lines. We propose that FOXM1 could be a potential therapeutic target for SS.

We also observed that in two SS cell lines, thiostrepton, known as a FOXM1 inhibitor [47], reduced both the number of viable cells in a dose-dependent manner and the levels of FOXM1 protein and mRNA expression. Reduced FOXM1 expression in protein and mRNA levels was recognized at low-toxic concentrations of thiostrepton in the HS-SY-II cells (1 μM), and the interruption of FOXM1 could not decrease the cell proliferation in HS-SY-II cells. It was contrary that recognized in SYO-1. This deference effect of FOXM1 interruption between the two cell lines, suggested that the FOXM1 involved in SS tumor progression in a variety of ways. We supposed that the difference might be due to the morphology (biphasic or monophasic) and genetic basement (SYT-SSX1 or SSX2). But there was no supportive finding by immunohistochemical study and cDNA microarray analysis.

The cytotoxicity in SS cell lines might be not only via the inhibition of FOXM1. The mechanism of FOXM1 interruption by thiostrepton has been proposed to be via the direct binding of FOXM1 [47] and also via its activity as a proteasome inhibitor [48].

Another proteasome inhibitor, bortezomib, also showed the ability to interrupt FOXM1, although there is no evidence of its direct binding to FOXM1 [48]. Little is known about the efficacy of proteasome inhibitors in SS. The proteasome inhibitor MG132 has shown antitumor activity for SS cell lines in vitro [49], However, the results from a Phase II trial of a single use of bortezomib against a variety of relapsed or metastatic sarcomas including SS have been discouraging [50]. The efficacy of combination conventional chemotherapy with a proteasome inhibitor against sarcoma has not been established. In our study, both cell lines treated with the combination of thiostrepton and DOX showed lower cellular proliferation than those treated with either drug individually. Thiostrepton has the potential to be a therapeutic agent for SS cases showing FOXM1 expression.

## Conclusion

We have elucidated that FOXM1 inhibition is a candidate treatment option for SS, based on our clinicopathologic assessment and in vitro study, using siRNA and thiostrepton on two SS cell lines. FOXM1 may be involved in SS tumor progression in a variety of ways. Further in vivo and in vitro investigations are warranted to evaluate the efficacy of FOXM1 inhibitors either alone or in combination with other agents.

## Abbreviations

AJCC, American Joint Committee on Cancer; DOX, doxorubicin; EFS, event-free survival; FNCLCC, French Federation of Cancer Centers; FOXM1, forkhead box M1; HPF, high-power fields; OS, overall survival; SS, synovial sarcoma; WHO, World Health Organization

## Additional files

Additional file 1: SS cell lines treated with 1 μM thiostrepton for 48 h. The real-time quantitative PCR showed a reduction of FOXM1 transcript. (PPTX 73 kb)
