# Supplementary material for: Prognostic significance of FOXM1 expression and antitumor effect of FOXM1 inhibition in synovial sarcomas
Source: BMC Cancer. 2016 Jul 20;16:511. doi: 10.1186/s12885-016-2542-4 (PMC4955131; doi:10.1186/s12885-016-2542-4)
Supplement: Additional file 1: — SS cell lines treated with 1 μM thiostrepton for 48 h. The real-time quantitative PCR showed a reduction of FOXM1 transcript. (PPTX 73 kb) [file 12885_2016_2542_MOESM1_ESM.pptx]

## Slide 1
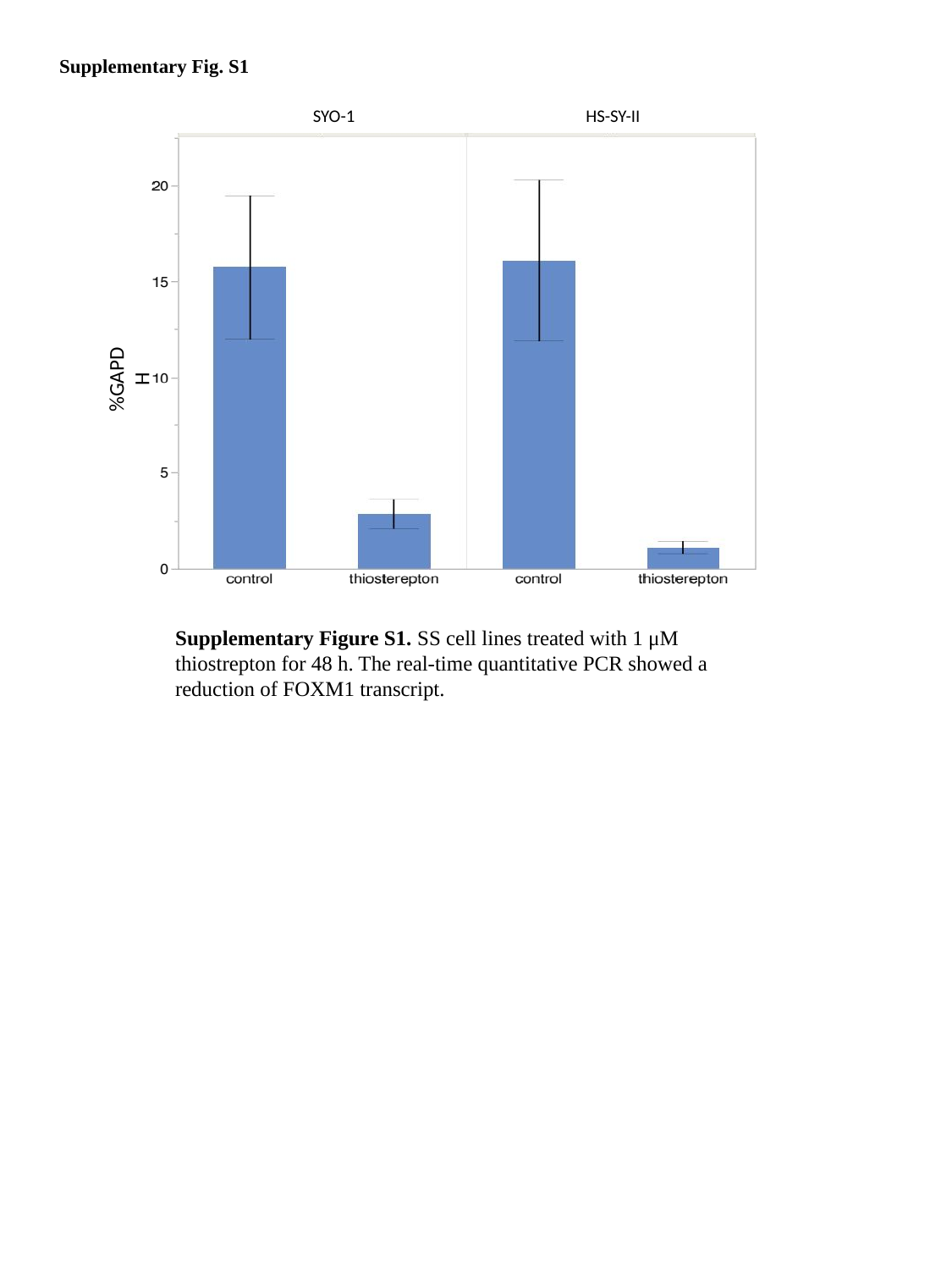

Supplementary Fig. S1
SYO-1
HS-SY-II
%GAPDH
Supplementary Figure S1. SS cell lines treated with 1 μM thiostrepton for 48 h. The real-time quantitative PCR showed a reduction of FOXM1 transcript.
